# Alterations in Lipids and Adipocyte Hormones in Female-to-Male Transsexuals

**DOI:** 10.1155/2010/945053

**Published:** 2010-07-18

**Authors:** Prakash Chandra, Sukhdeep S. Basra, Tai C. Chen, Vin Tangpricha

**Affiliations:** ^1^Division of Endocrinology, Metabolism and Lipids, Department of Medicine, Woodruff Memorial Research Building, Room 1301, 101 Woodruff Circle NE, Atlanta, GA 30322, USA; ^2^School of Public Health, University of Texas at Houston, Houston, TX 77030, USA; ^3^Section of Endocrinology, Diabetes and Nutrition, Department of Medicine, Boston University School of Medicine, Boston, MA 02118, USA

## Abstract

Testosterone therapy in men and women results in decreased high-density lipoprotein cholesterol (HDL) and increased low-density lipoprotein cholesterol (LDL). We sought to determine whether testosterone therapy has this same effect on lipid parameters and adipocyte hormones in female-to-male (FTM) transsexuals. Twelve FTM transsexuals provided a fasting lipid profile including serum total cholesterol, HDL, LDL, and triglycerides prior to and after 1 year of testosterone therapy (testosterone enanthate or cypionate 50–125 mg IM every two weeks). Subjects experienced a significant decrease in mean serum HDL (52 ± 11 to 40 ± 7 mg/dL) (*P* < .001). The mean LDL (*P* = .316), triglyceride (*P* = .910), and total cholesterol (*P* = .769) levels remained unchanged. In a subset of subjects, we measured serum leptin levels which were reduced by 25% but did not reach statistical significance (*P* = .181) while resistin levels remained unchanged. We conclude that testosterone therapy in FTM transsexuals can promote an increased atherogenic lipid profile by lowering HDL and possibly reduce serum leptin levels. However, long-term studies are needed to determine whether decreases in HDL result in adverse cardiovascular outcomes.

## 1. Introduction

Transsexualism is a medical condition characterized by gender dysphoria which is managed with cross-sex hormones and surgery to align an individual's physical appearance with their gender orientation [1, 2]. Female-to-male (FTM) transsexuals take testosterone to change into a more masculine phenotype. Current guidelines remain uncertain about long effects of testosterone therapy on cardiovascular outcomes due to lack of long-term studies and suggest risk factors including lipids to be managed individually [1]. A recent meta-analysis of ten studies of testosterone therapy in FTM transsexuals by Elamin et al. demonstrated increased serum triglycerides (TG) and reduced high-density lipoprotein (HDL) concentrations. However, the data remained unclear about testosterone's effects on other lipid fractions and blood pressure [3]. Other studies not examined in the meta-analysis by Elamin et al. have demonstrated increased total cholesterol (TC) [4] and low-density lipoprotein (LDL) levels [4, 5] in FTM transsexuals receiving testosterone therapy. Similarly, 17 FTM transsexuals treated with intramuscular injections of long acting testosterone undecanonate for 36 months demonstrated a decrease in TC and LDL without effecting triglyceride and HDL levels [6]. 

Gooren et al. [7] suggested that an increased atherogenic lipid profile can be a result increased weight and visceral fat after testosterone therapy in FTM transsexuals [8]. This changed adiposity pattern can lead to differential adipocyte hormone secretion [9, 10]. However, studies evaluating the effect of testosterone therapy on adipocyte hormones in FTM transsexuals are minimal and available data remain inconclusive. While Elbers et al. [11] reported supraphysiological doses of testosterone in FTM to reduce leptin levels; others have failed to confirm this finding [12]. Similarly, the effect of supraphysiologic doses of testosterone administration on resistin still remains to be understood though endogenous androgens in Polycystic Ovarian Syndrome (PCOS) have been shown to cause elevated resistin levels after controlling for body mass [13].

We hypothesized that supraphysiologic (male replacement) doses of testosterone in FTM transsexuals (genetic females) would lead to a more atherogenic lipid profile and increase markers of insulin resistance. Our prospective clinical trial evaluated changes in the lipid profile and the adipokines, resistin and leptin, in FTM transsexuals before and after treatment with male-replacement doses of testosterone.

## 2. Methods

The study was approved by the Boston University School of Medicine IRB and was conducted at the Boston University General Clinical Research Clinic (GCRC).

### 2.1. Study Subjects

Subjects were recruited from the endocrinology clinics at Boston University as previously described [14]. Briefly, all subjects were genetic females who were deemed to have gender identity disorder by their mental health provider and were recommended for treatment with testosterone therapy. For this study, we evaluated subjects who had not previously received testosterone therapy and were analyzed prior to sex reversal surgery.

### 2.2. Procedures

We prospectively monitored the serum lipoprotein profile of 12 FTM transsexuals who were started on supraphysiologic doses of testosterone esters, cypionate, or enanthate (supraphysiologic for a female but replacement levels for a male) (50–125 mg every two weeks to attain male physiological levels of serum testosterone), for 12 months. Subjects provided a fasting blood sample for TC, LDL, LDL, HDL, and triglyceride measurements at baseline and 1 year after initiating testosterone therapy. Due to sample availability, we measured serum resistin and leptin levels only in a subset of subjects (*n* = 5) at baseline and at least 6 to 12 months after initiation of testosterone therapy.

Cholesterol, glucose, hematocrit, and total testosterone were measured by a commercial laboratory (Quest Diagnostics, (San Juan Capistrano, CA). The resistin and leptin levels were drawn at the same time as the testosterone and at the “trough” level of testosterone. Leptin and Resistin were measured using commercially available ELISA kits from ALPCO Diagnostics (Salem, New Hampshire). The intra- and inter-assay CVs for the Leptin ELISA were 3.7%–5.5% and 5.8%–6.9%, respectively. The intra- and inter-assay CVs for the resistin ELISA were 2.9%–5.2% and 4.2%–7.2%, respectively.

### 2.3. Statistical Analysis

The results were represented as means ± SEM. The data were analyzed using Microsoft Excel (Office 2000) and Sigma Stat Version 3.0. Differences in levels of serum lipid levels were evaluated by using paired *t*-test. Differences in serum resistin and leptin levels were evaluated by mean percentage change from baseline.

## 3. Results

### 3.1. Subject Demographics

Table 1 demonstrates the baseline demographics of the study population. The mean age of our subjects was 29 ± 9 years. Ten out of 12 subjects identified themselves as of Caucasian ethnicity, while 1 each were from Hispanic and African American ethnicity. Four subjects identified themselves as active smokers.

### 3.2. Changes in Clinical and Biochemical Markers in Response to Testosterone

As expected, serum testosterone values increased after the initiation of testosterone therapy (*P* < .001) (Table 2). The increase in testosterone was accompanied by an increase in hematocrit (*P* < .001) (Table 2). We did not observe any changes in the clinical parameters of body mass index and mean arterial pressure or in the biochemical parameters of the liver transaminases (SGOT/SGPT) or platelet count (Table 2).

### 3.3. Changes in Lipid Parameters

We observed a significant decrease in high-density lipoprotein (HDL) cholesterol (*P* < .001) but no significant changes in the other lipid parameters such as low-density lipoprotein (LDL), total cholesterol, or triglycerides (Table 2). The serum resistin and leptin (*n* = 5) remained unchanged from 10.3 ± 2.1 to 9.6 ± 2.2 ng/dL (*P* = .679) and 31.2 ± 29.5 to 19.1 ± 17.5 ng/dL (a 25% reduction, *P* = .181), respectively (Table 2).

## 4. Discussion

Our study demonstrated a significant decrease in the concentration of HDL after 1 year of testosterone therapy in FTM transsexuals, as previously reported by other studies [3, 4, 15]. The serum LDL, TG, and TC did not show a significant change. The leptin and resistin levels did not demonstrate a significant change, owing to the small number of subjects. There was no increase in mean arterial blood pressure or change in BMI after two years of therapy. Liver enzymes and fasting blood glucose levels remained unchanged, while as expected there was a rise in hematocrit.

 Several studies have examined the effects of testosterone on lipid concentrations in females and in FTM transsexuals. Goh et al. demonstrated a decrease in HDL and an increase in LDL, TG, and TC levels after long-term testosterone therapy in women [4]. Mueller et al. demonstrated an increased serum TG concentration and a decreased HDL concentration without any change in LDL in FTM subjects treated with testosterone [15]. Similarly, Elbers et al. demonstrated a decreased HDL and LDL particle size and increased TG concentration after testosterone administration in FTM transsexuals [8]. In contrast, Jacobeit et al. [16] demonstrated that testosterone therapy in FTM transsexuals resulted in a statistically significant decrease in the level of TC and LDL, with no change in HDL. A recent meta-analysis of testosterone therapy in FTM transsexuals demonstrated an increase in TG and decrease in HDL in FTM transsexuals [3]. Of importance, the increase in TG levels was more significant in studies which had longer followup times [3]. Our study is consistent with the majority of studies that demonstrate a reduced HDL level but we were unable to demonstrate a change in TG levels likely due to the short-term nature of our study. 

 Despite seemingly adverse changes in the lipid profile from testosterone administration in FTM transsexuals, testosterone administration has not been associated with increased cardiovascular morbidity or mortality in FTM transsexuals [3, 7]. Therefore, in a subset of our patients, we investigated effects of testosterone supplementation on adipocyte hormones that are putative cardiovascular risk factors. Leptin is an adipocyte hormone that signals receptors in hypothalamus to decrease food intake and stimulates energy expenditure [17]. Studies have demonstrated leptin to be an independent risk factor for stroke and myocardial infarction after controlling for obesity status [18, 19]. Leptin administration has been reported to raise serum TG, LDL, and TC levels [20]. Supraphysiological doses of testosterone in FTM transsexuals have been reported to reduce plasma leptin concentration [11, 21]. However, Resmini et al. [12] reported a strong interindividual variability in response of plasma leptin levels to testosterone therapy and, hence, failed to show a significant posttreatment change. Similarly, in a subset of our subjects, we found a 25% decrease in plasma leptin concentration with testosterone therapy which did not reach statistical significance likely to the small number of subjects evaluated. Therefore, we can only conclude that larger and more long-term studies need to be conducted to confirm the effects of testosterone on leptin concentrations in FTM transsexuals.

Resistin is an adipocytokine that is associated with obesity-mediated insulin resistance [22] and increased cardiovascular risk [23]. We are not aware of a previously published study to evaluate testosterone therapy on resistin concentrations in FTM transsexuals. Data from females with elevated endogenous androgen levels as occurring in PCOS have attempted to evaluate the relationship between resistin and androgen concentrations. Seow et al. found no difference in serum resistin levels in patients with and without PCOS and there was no correlation between serum resistin and testosterone levels [24]. However in contrast, Carmina et al. reported elevated plasma resistin levels in PCOS patients [13]. Finally, Yilmaz et al. [25] reported that plasma resistin levels were not associated with androgen concentrations in women with PCOS [25]. In summary, few studies have evaluated the relationship between endogenous testosterone levels or testosterone therapy on resistin concentrations. Our study did not demonstrate any significant differences in the resistin levels after administration of testosterone in FTM transsexuals.

## 5. Conclusion

Testosterone therapy in FTM transsexuals results in a more atherogenic lipid profile by significantly decreasing HDL. Although limited by the population size, our preliminary study found that initiation of testosterone therapy in female-to-male (FTM) transsexuals may lower plasma leptin levels. Long-term studies are needed to monitor cardiovascular events in FTM transsexuals on testosterone therapy to determine if these changes in the lipid profile and adipocyte hormones translate into increased cardiovascular events.

##  Conflict of Interest

The authors report no conflict of interest.

## Figures and Tables

**Table 1:** Demographic background of female to male transsexuals prior to the initiation of testosterone therapy (*n* = 12).

	*N* = 12
Age (years)	29 ± 9
Race (*n*) African/Caucasian/Hispanic	1/10/1
Smokers (*n*)	4

**Table 2:** Biochemical changes in response to 12 months of testosterone therapy in female-to-male transsexuals, including changes in lipid profile and markers of insulin resistance. Mean ± SEM, *N* = 12 unless specified.

	Prior to Testosterone	12 months	*P*-value
Body Mass Index (BMI) (kg/m^2^)	27.5 ± 5.2	27.6 ± 3.9	.47

Mean Arterial Pressure (MAP (mm Hg)	87 ± 14	91 ± 16	.16

Serum Glutamic Oxaloacetic Transaminase (SGOT) (IU/L)	21 ± 5	25 ± 7	.22

Serum Glutamate Pyruvate Transaminase (SGPT) (IU/L)	19 ± 7	24 ± 10	.22

Serum Total Testosterone (nmol/L)	3.4 ± 3.0	34.6 ± 8.5	<.001

Hematocrit (%)	40 ± 2	45 ± 5	<.001

Platelets (×1000/*μ*L)	282 ± 44	266 ± 38	.21

High Density Lipoprotein (HDL) (mg/dL)	52 ± 11	40 ± 7	<.001

Low Density Lipoprotein (LDL) (mg/dL)	113 ± 22	121 ± 29	.32

Total Cholesterol (mg/dL)	184 ± 26	181 ± 34	.77

Triglycerides (mg/dL)	92 ± 72	94 ± 41	.91

Leptin (ng/dL)	31.2 ± 29.5	19.1 ± 17.5	.18

Resistin (ng/dL)	10.3 ± 2.1	9.6 ± 2.2	.68

Fasting glucose (mg/dL)	86 ± 12	83 ± 11	.43
